# Bruxism’s Implications on Fixed Orthodontic Retainer Adhesion

**DOI:** 10.3390/dj10080141

**Published:** 2022-08-01

**Authors:** Anca Labuneț, Adriana Objelean, Oana Almășan, Andreea Kui, Smaranda Buduru, Sorina Sava

**Affiliations:** 1 Prosthetic Dentistry and Dental Materials Department, Iuliu Hațieganu University of Medicine and Pharmacy, 31 Avram Iancu Street, 400083 Cluj-Napoca, Romania; labunet@yahoo.com (A.L.); adriana.caracostea@gmail.com (A.O.); savasorina@yahoo.com (S.S.); 2 Prosthetic Dentistry and Dental Materials Department, Iuliu Hațieganu University of Medicine and Pharmacy, 32 Clinicilor Street, 400006 Cluj-Napoca, Romania; andreeakui@gmail.com (A.K.); smarandabudurudana@gmail.com (S.B.)

**Keywords:** bruxism, adhesion, orthodontics, fixed retention

## Abstract

Background: Fixed retainers assist in maintaining the outcomes of orthodontic treatment. Fixed retention may be affected by bruxism. Objective: Evaluate two adhesives (an ormocer and a flowable composite) used for fixed orthodontic retention in simulated bruxism settings, compared to regular mastication, using a dual axis chewing simulator. Methods: Eighty human teeth were used. Periodontal tissues were simulated and exposed to 120,000 mechanical cycles, corresponding to 6 months of clinical service. Each set of two teeth was supplied with a pre-shaped, fixed, multi-braided, stainless steel wire retainer, in 1.5 cm portions, to establish passive contact with the lingual surface of the teeth. The Adhesive Remnant Index (ARI) was used to evaluate the shear bond strength. A stereomicroscope was used to assess the micro-infiltration. Results: There was no significant difference in the mean value of micro-infiltration between adhesives in the mastication group but in the bruxism group. During testing, one composite sample (ARI score 1) was broken in the mastication group, while three ormocer samples (ARI score 2) and one composite sample (ARI score 1) were broken in the bruxism group. Conclusions: The mean value for micro-infiltration in composite (0.31) was more than double that in ormocer (0.13).

## 1. Introduction

Retention of the orthodontic results is considered a key element for orthodontic treatment to be successful over the long term because it will keep the teeth in their ideal position after therapy. Adhered retainers may be crucial in preventing unintentional tooth movements after orthodontic therapy [1]. Orthodontists frequently recommend either removable or fixed retainers to aid patients to maintain the treatment results [2]. Typically, fixed retainers are preferred by orthodontists over removable ones as they are more aesthetically pleasing, require less patient cooperation, and are suitable for long-term retention [2,3]. Even fixed retainers have a chance of failure due to wire breakage or separation between the wire and the adhesive interface. The most frequent type of collapse seen in fixed lingual retainers is an adhesive failure, with no influence of the wire type on the failure rate [4]. As a result, the fixed retainer’s fracture or debonding could trigger a recurrence of the orthodontic issue [3,5]. In terms of bond strength, according to Golshah and Amiri Simkooei, there is no advantage between wire retainers with a round cross-section and those with a flat, rectangular-shaped cross-section [6].

Along with stainless steel retainers, nano-apatite grafted glass-fiber-reinforced composites are used as orthodontic retainers, showing comparable bonding force [7].

Bruxism is a major concern for dentists because it can have a detrimental effect, including damage to dental restorations or prosthetic rehabilitations, headaches that may or may not be related to temporomandibular disorders (TMDs), and tooth wear [8].

Although there has been controversy over the definition of bruxism for several years. a global consensus on the subject was reached in 2013 and is now accepted as follows: “bruxism is a repetitive masticatory muscle activity that is characterized by clenching or grinding of the teeth and/or by bracing or thrusting of the mandible, and that is specified as either sleep bruxism or awake bruxism, depending on its circadian phenotype” [9,10]. The etiology of bruxism includes both psychological and local factors, such as dental malocclusion, as well as psychological factors, such as stress, systemic diseases, trauma, and sleep disorders. There is currently no single treatment that would successfully overcome and reduce bruxism because there is not a single cause responsible for bruxism [11].

Orthodontists are also concerned about bruxism since it puts the dental-maxillary structures under parafunctional stress. Additionally, due to high mechanical stress and additional forces put on the bonded teeth by bruxism, fixed orthodontic retainers may fail [2,8]. On the other hand, the effect of orthodontic retainers on healthy people’s masticatory muscle activity during sleep has been investigated, showing no noticeable effects [12]. To reduce the negative effects of sleep bruxism, various oral appliances have been suggested: metal and resin bites have been shown to reduce the sleep bruxism index, whilst clear aligners have no effect on the sleep bruxism index, but reduce muscle contractions [13].

Concerning bruxism’s effects when receiving orthodontic treatment, there is little evidence published in the literature. Therefore, this study’s main objective was to assess microleakage for two adhesives used as fixed orthodontic retainers in simulated bruxism conditions and simulated normal mastication conditions, and compare the results. The null hypothesis stated that there is no difference in microleakage under normal mastication and bruxism conditions, as simulated on extracted teeth bonded with a fixed retainer wire using either composite or ormocer, and that there is also no difference between composite and ormocer in terms of microleakage.

## 2. Materials and Methods

**Figure 1:**
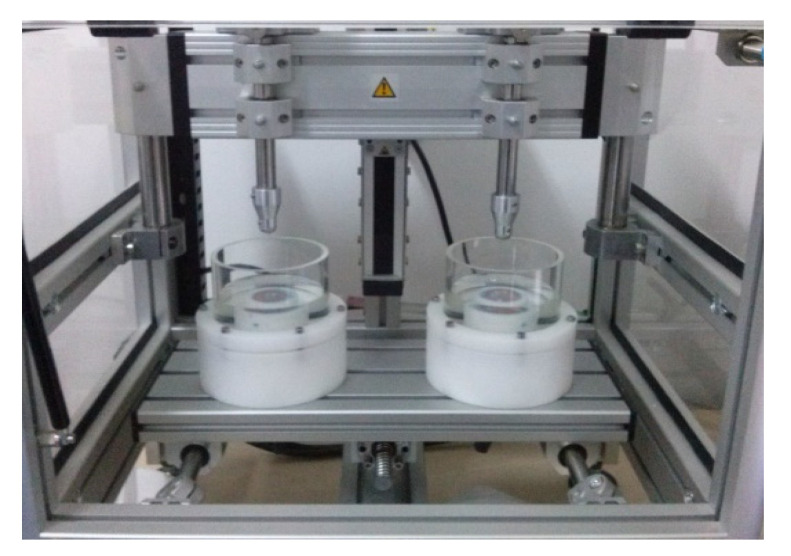
Dual-axis chewing simulator (CS-4.2, SD Mechatronik, Germany).

This in vitro study was conducted between June 2019 and July 2020, in the Dental Materials Department at the University of Medicine and Pharmacy “Iuliu Hațieganu” in Cluj-Napoca. The study was approved by the university’s ethics committee (approval number 805.13.05.26). The teeth were collected from the surgery department of our university. Human lower incisors that had been removed for orthodontic or periodontal reasons were chosen. The teeth were healthy and had no caries lesions. Using a dual-axis chewing simulator (CS-4.2, SD Mechatronik, Feldkirchen-Westerham, Germany; Figure 1) to replicate normal masticatory conditions and bruxism conditions, teeth were tested for two different materials: a flowable composite, Gradia direct flo^®^ (GC, Tokyo, Japan) and an ormocer, Admira^®^ (VOCO, Cuxhaven, Germany). A total of eighty teeth were enrolled. Four groups were formed, each containing twenty teeth: the first group was the composite group, under mastication conditions, (1; Gradia—mastication conditions), the second group was the ormocer group under mastication conditions (2; Admira—mastication conditions); the third group was the composite group under bruxism conditions (3; Gradia—bruxism conditions) and the fourth group was the ormocer group under bruxism conditions (4; Admira—bruxism conditions).

### 2.1. Preparation of Teeth

All teeth included in the research were carefully cleaned for 2–5 min with an ultrasound device (Woodpecker Handpiece, Guilin, China), and then polished for 3 min with a fluoride-free pumice paste (Proxyt RDA 36 Ivoclair, Schaan, Liechtenstein). Teeth were continuously kept in a 9 percent saline solution until they were further bonded.

### 2.2. Preparation of Samples

The periodontal tissues were also simulated for accurate replication of intraoral conditions, using a technique described by Brosh et al. [14]. The roots of the teeth were first covered with a thin layer of wax. After that, the teeth were paired and placed inside Duracryl disks made of acrylic resin. The wax layer was detached, and the teeth were removed from the resin after it had set. A light body, addition-cured silicone was injected before each tooth was reinserted into the orifice that had been created in the acrylic resin. In this manner, the periodontal ligaments would be mimicked after the silicone coat had dried, assuring similar elasticity. Using multi-braided stainless steel wire cut into 1.5-cm sections and pre-shaped to have passive contact with the teeth’s lingual surface, each pair of two teeth received fixed orthodontic retention. According to the producer’s indications, one of the two adhesives considered for this in vitro study was used to bond the wire. The Adhesive Remnant Index (ARI), as described by Leodido et al., was used to evaluate the shear bond strength [15]. According to this classification, an ARI index of 0 was used when no adhesive remained on the enamel; an ARI index of 1 when less than 50% of the adhesive remained on the enamel; an ARI index of 2 when more than 50% of the adhesive remained on the enamel, and an ARI index of 3 when all adhesive remained on the enamel [15].

A stereomicroscope with 4 and 40 times magnification was used to assess the micro-infiltration. One millimeter (mm) sections of the composite and ormocer samples were analyzed.

The preparation of the composite samples (Gradia groups) was as follows: after etching with orthophosphoric acid 37% for 30 s, rinsing for 10 s, and drying, a two-step etch-and-rinse adhesive system Optibond Solo Plus (Kerr/Sybron, Orange, CA, USA) was applied for 20 s and then light-cured (using a halogen curing lamp called the Optilux 501, made by Kerr/Demetron, Brea, CA, USA) for an additional 20 s. Afterward, when the stainless steel retainer was put in place (multi-braided stainless steel, G&H Orthodontics, Franklin, IN, USA), the composite flowable paste was applied and light-cured for at least 20 s on each tooth.

The process of preparing the ormocer samples (Admira groups) comprised the following steps: orthophosphoric acid (Bisco, Lombard, IL, USA) at a concentration of 37% was applied for 30 s, rinsed for 10, and dried for 10 s. For each tooth, Admira Bond (VOCO, Cuxhaven, Germany) was applied for 20 s, light-cured for 20 s, followed by the application of the metallic wire (multi-braided stainless steel, G&H Orthodontics, Franklin, IN, USA) and the flowable ormocer paste.

**Figure 2:**
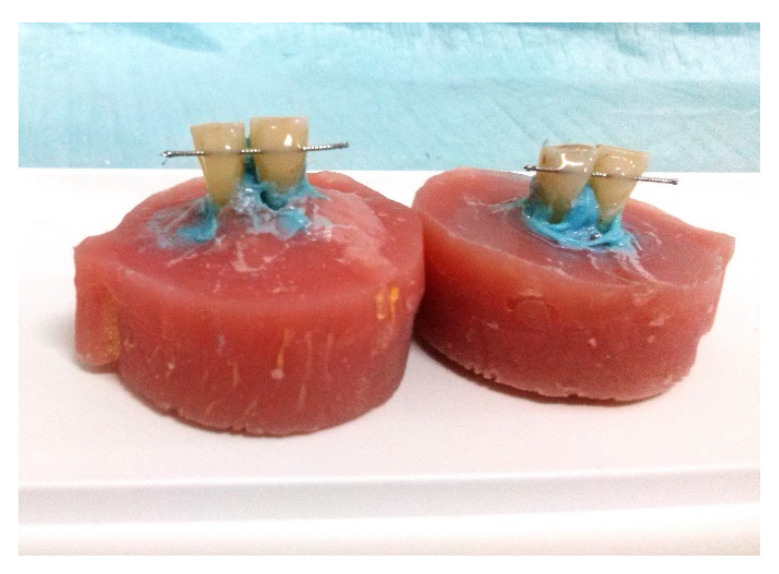
The prepared samples: composite group and ormocer group.

The prepared samples are shown in Figure 2.

### 2.3. Testing the Samples

**Figure 3:**
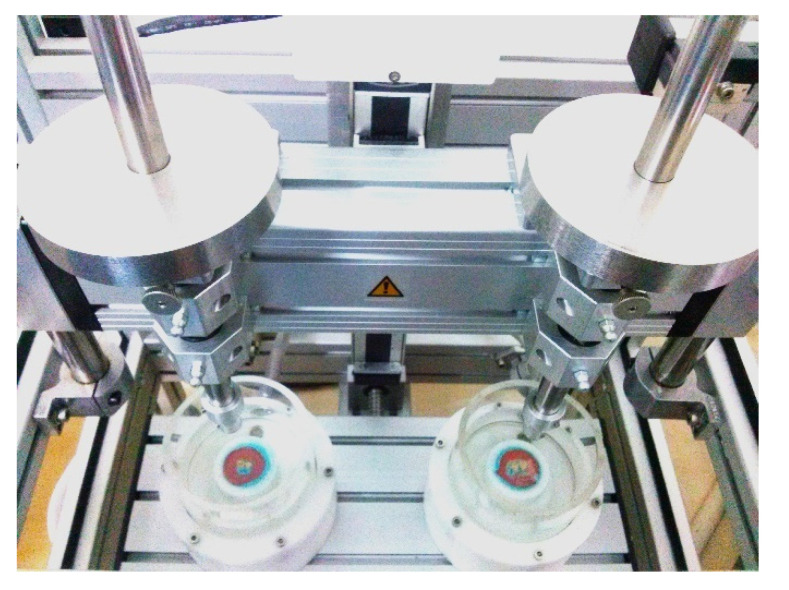
The testing of the samples using a dual-axis chewing simulator.

A dual-axis chewing simulator was used to test each sample (Figure 3) (CS-4.2, SD Mechatronic, Feldkirchen-Westerham, Germany). This kind of simulator performs well in various testing methodologies [9]. At the occlusal-proximal contact of the teeth, ceramic styli in the shape of cusps were placed.

To simulate mastication conditions: the teeth underwent 120,000 mechanical cycles, which correspond to six months of clinical service at 1 kgf at a frequency of 1.6 Hz, with lateral travel of 0.7 mm. Each sample was immersed in distilled water during testing [16,17].

To simulate bruxism conditions: the teeth underwent 120,000 mechanical cycles or six months of clinical service at 5 kgf 1.7 Hz, and 1.5 mm lateral movement. Each sample was also submerged in distilled water throughout the testing process [16].

### 2.4. Sample Analysis 

Following testing in the chewing simulator, the samples from the two materials used in this in vitro study were submerged for 24 h in a 2 percent basic fuchsine solution to assess their marginal integrity. The samples were then rinsed and buccolingual sectioned with a low-speed saw (IsoMet 1000, Buehler Ltd., Lake Bluff, IL, USA). Using an Olympus KC-301 stereomicroscope (Olympus America Inc., Center Valley, PA, USA) at 4× and 40× magnification, a 1 mm section of each tooth was taken, and the tracer’s infiltration was further examined. The micro-infiltration was then verified using the provided Quick Photo Micro 2.2 software (Olympus America Inc., Center Valley, PA, USA).

### 2.5. Statistical Analysis

We determined the sample size for this study using a previous pilot study, in which the effect size of 1 was added to a power (1 − b) = 0.95 and a level of significance of a = 0.3. We analyzed the data into a procedure of a *t*-test family (Paired-samples *t*-test) using G*power 3.1 (Heinrich-Heine-University Software, Düsseldorf, Germany) for Windows software. The optimal sample size was calculated up to 10 statistical pairs.

Using the statistical analysis software SPSS 22.0 (Statistical Package for Social Sciences software 22.0—SPSS, Chicago, IL, USA), all the collected data were analyzed. Descriptive statistics were used as a preliminary step. The data distribution was then ascertained by the Kolmogorov–Smirnov test. Due to the non-normal distribution of the data, Mann–Whitney tests were used. The U test was used to evaluate the null hypothesis (*p* ≤ 0.05).

## 3. Results

**Figure 4:**
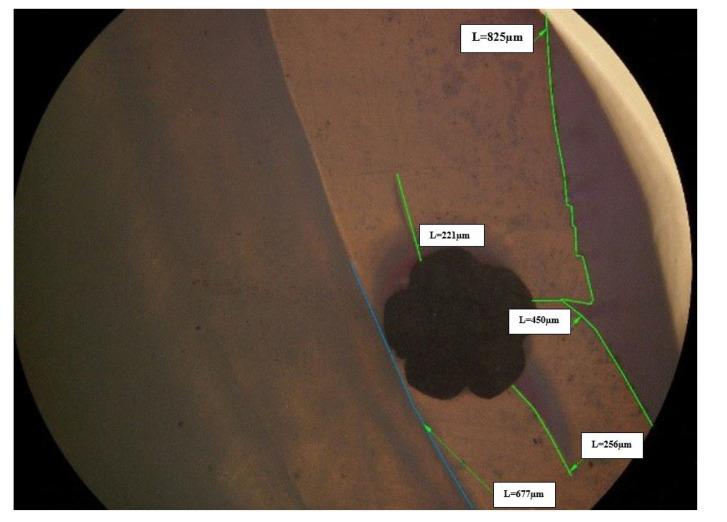
Stereomicroscopy at a magnification of 40 times of a 1 mm section of a composite specimen displaying the retainer-incorporating composite on the enamel surface.

**Figure 5:**
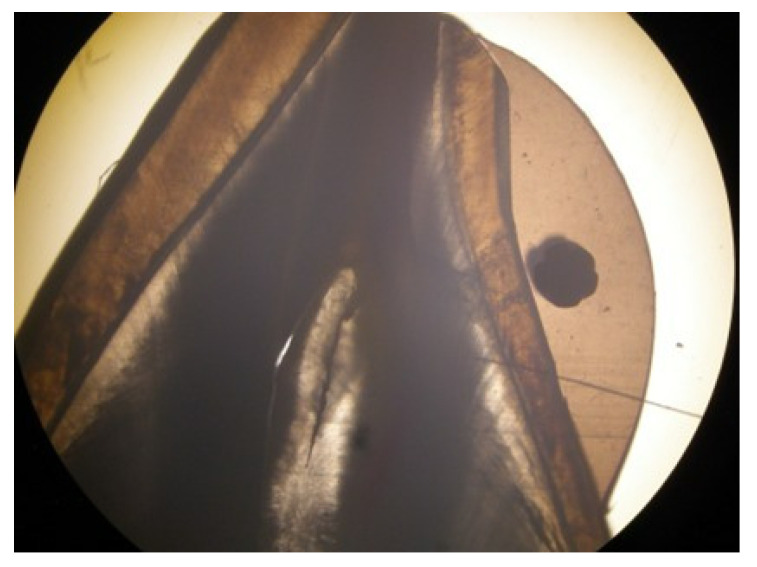
Stereomicroscopy at a magnification of 4 times of a 1 mm section of a composite specimen displaying the retainer-incorporating composite on the enamel surface.

For each one millimeter section, a ratio was calculated by comparing all the measured values for the micro-infiltration (expressed in micrometers) with the total interface between the tested material and enamel. The 40 times magnification stereomicroscopy of a 1 mm section of a composite specimen displaying the retainer-incorporating composite on the enamel surface is shown in Figure 4, and the 4 times magnification of the same sample is shown in Figure 5.

Two specimens from the bruxism group and two more from the mastication group were excluded from further analysis because they had no micro-infiltration (Table 1). Three ormocer specimens and one composite specimen broke during testing in the bruxism group, while one composite specimen broke during testing in the mastication group. Mann–Whitney U test significance (2-tailed value) for the mastication group was 0.068. The value in this group prevented us from ruling out the null hypothesis. The group statistics showed that the mean value of microscopic infiltration between the composite and ormocer specimens in the mastication group did not differ significantly (Table 2).

The significance (2-tailed) value of the t-test for the bruxism group was 0.042, which indicates that the null hypothesis was rejected, and that there was a statistical difference between the composite and ormocer specimens. The group statistics revealed that the composite (Gradia) specimens’ mean micro-infiltration value (0.31) was more than twice as high as the ormocer (Admira) value (0.13), as shown in Table 3.

The Adhesive Remnant Index (ARI) for the composite specimen that broke in the mastication group was 1, while the ARI for the three ormocer broken specimens in the bruxism group was 2, and the ARI for the composite broken specimen was 1.

Additionally, we compared the ormocer samples between the two groups (bruxism and mastication), and the results revealed a Mann–Whitney U test significance value of 0.380 (*p* < 0.05). The statistical analysis of the composite specimens in the two groups generated a t-test significance value of 0.542 (*p* < 0.05).

## 4. Discussion

Lifelong retention is frequently advised after orthodontic treatment because patients who receive orthodontic treatment anticipate having a stable occlusion for the rest of their lives [18,19]. However, fixed orthodontic retention can also fail or even have adverse effects. In choosing the fixed retention protocol, special attention must be given to the bonding agent as well as to the wires’ material properties and composition.

This study’s objective was to assess the microleakage of two materials used to bond a fixed retainer under simulated chewing and bruxism conditions. The practitioners and other researchers currently use both tested materials for bonding fixed retainers. According to some studies, ormocer performs well to bond orthodontic brackets and is also more biocompatible, and has a lower wear rate than other adhesives [20,21], which is consistent with our findings.

Ormocer has been shown to have some advantages, including minimal micro-infiltration and polymerization shrinkage as well as superior biocompatibility due to the sheer minimal monomer content [22,23,24].

For all samples, we have used the etch-and-rinse method of phosphoric acid etching of enamel, although nowadays many self-etch adhesive systems are in use. As recommended by the authors Erickson et al., the bond produced by etch-and-rinse systems using phosphoric acid etching of enamel cannot be competed with, despite chemical bonding possibly being present in some self-etch adhesive systems [25].

All teeth that were included in the study were healthy, with no caries lesions. Extra care was given to operate in a contaminant-free environment. According to Campanella et al., the type of dentin affects the performance of modern adhesive systems, the clinical effectiveness of bonding being influenced by the variability of the dentin substrate: normal dentin, caries-affected, or dentin contaminated by metallic oxides [26].

Jedliński et al., evaluated fixed retainer retention procedures and materials for orthodontic retainers and found that the most reliable wire was the rectangular steel braided wire, however, the most commonly used material is stainless steel braided wire, bonded with flowable composite; and fiber-reinforced composite is being used in periodontal patients [27]. In our study, we have used pre-shaped, multi-braided, stainless steel wire, which was constructed into passive contact with the teeth’s lingual surface, bonded with a flowable composite or an ormocer.

Paolone et al., tested the performance of different lingual orthodontic retainers bonded with composite a straight wire, two round twisted wires, and a rectangular braided wire, and showed that the bonding between wires and composites appeared to be weakest for rectangular smooth wires and strongest for round twisted and rectangular twisted wires [28]. Regarding the bonding agents, hybrid composites had the lowest interface bonding values, whereas nano- and micro-composites could withstand greater forces and had higher bonding values [28].

Al-Nimri and Al-Nimri compared the bond strength of different wire diameters fixed orthodontic retainers using a conventional composite and a specific retainer composite, and showed that the failure site wasn’t associated with the wire’s diameter or the adhesive, however, using a specific retainer composite and a decreased wire diameter showed to have an increased bond strength [29].

Tabrizi et al., have evaluated different types of composites for bonding orthodontic retainers and highlighted the fact that flowable composites can be used as an alternative for bonding lingual retainers [30].

Uysal et al. have tested the suitability of flowable composites in orthodontic bracket bonding and showed that the bond strength of flowable composites was lower when compared to an orthodontic bonding agent, and also evaluated the ARI index scores of the testes samples [31]. However, the authors tested the bond strength of bracket bonding and did not use bruxism conditions. Pick et al. also compared the bond strength and adhesive remnant index of flowable resin-based composites and orthodontic adhesive systems for metal bracket bonding, and showed that unless the teeth to be bonded are not subjected to higher orthodontic stresses, flowable composites may not be suitable for bracket bonding [32].

In testing shear bond strength, various authors evaluated different composites for fixed orthodontic retainer bonding. In this study, we have used also an ormocer, which was not reported as a fixed orthodontic retainer bonding agent.

One of the main axioms in orthodontics that should not be changed to maintain the treatment’s results is the intercanine width. Therefore, in clinical settings, the bonding of fixed retainers from canine to canine is pursued. A study using rigid and flexible lingual retainers to assess the three-dimensional analysis of the post-treatment displacements of mandibular anterior teeth, using either rigid retainers bonded only to canines or a flexible stainless steel retainer bonded to all six mandibular teeth, showed that particularly in sagittal and transverse dimensions, central incisor contacts were more likely to shift with the rigid retainers [33]. Other authors studied the resistance of conventionally bonded mandibular retainers in patients with orthodontic treatment, either with or without enamel sandblasting, and found no discernible difference between the failure rates of mandibular bonded retainers with or without sandblasting. [34]. It has also been shown that intercanine and interpremolar distances could be maintained with bonded retainers [35]. Nevertheless, authors testing these scenarios used clinical situations, in which bonding has been performed on the six lower frontal teeth. However, in this study, we decided that we could benefit from only a small amount of testing space, roughly 5 cm for the base, for use with the mastication simulation device. The ceramic stylus that mimicked opposing teeth had a diameter of about 2 mm. Therefore, we could use only two teeth for testing purposes. We think that the two adjacent teeth should be the point of application of the force simulating bruxism to simulate retainer breakage. The testing of a larger number of bonded teeth was not possible with the utilized simulation device.

Comparing mean values between the composite group and the ormocer group in our study revealed higher micro-infiltration for the composite group. These findings appear to support the use of ormocer materials in clinical situations where biocompatibility and wear resistance are essential, as in the construction of bonding retainers. However, two specimens from the bruxism group and two from the mastication group showed no micro-infiltration. Chakraborty et al., in an vitro study, assessed the microleakage in bulk-fill composites, nanohybrid ormocer-based resins, and nano-filled composite resin, studied their core build-up materials employing the dye-penetration technique, and showed that all tested materials had micro leakage, with ormocer having the least [36]. Nevertheless, the authors did not test the bonding of orthodontic retainers, nor the bruxism simulated conditions.

During testing, three ormocer specimens and one composite specimen broke in the bruxism group while one composite specimen broke in the mastication group, showing the negative effect of bruxism on bond strength. Bruxism has negative effects not only on orthodontic fixed retention bond strength, but also on metal-wire reinforced composite bridges, showing an increased failure rate of those restorations [37]. Abreu et al. have evaluated the longevity of bulk-fill and ormocer composites in permanent posterior teeth, showing that nanofillers and nanohybrid resins exhibited better clinical longevity than ormocer composites in posterior restorations, but were similar to bulk-fill composites [38]. Considering the resistance of ormocers to occlusal forces, we settled upon testing an ormocer material compared to a flowable composite in testing the orthodontic bond strength of fixed retainers.

There are no actual studies examining the use of ormocer as a retainer bond under various masticatory conditions. A comparison between these conditions and various materials was required because it has been established that bruxism results in more significant tooth wear than normal mastication.

Tooth mobility is essential for building a realistic model when simulating fixed retention. Since the natural mobility of teeth may affect the bond strength of the retainer, periodontal tissues should be simulated. We used polyvinyl siloxane for periodontal ligament simulation in accordance with the study by Brosh et al. [14]. The teeth have been integrated with a substance that mimics periodontal tissues and can move within a rigid bone-like socket and transmit masticatory and bruxism forces.

Clinical observational studies have shown that the two most frequent reasons for fixed retention failure are wire breakage and adhesion failure [39]. Even though there is evidence that some types of retainers are less likely to break, some drawbacks include the difficulty of the technique and the fragility of the wires [40]. The separation of the tooth-adhesive interface is the most frequent type of failure [41].

In metal retainers, this failure type is reported to occur at a rate of 3.5–53%, whereas in fiber retainers, this rate changes from 11–51% [42,43]. Our study also demonstrated the failure of some samples. One composite specimen for the mastication group broke during testing, accruing an ARI score of 1, and three ormocer specimens and one composite specimen for the bruxism group both broke during testing. For the bruxism group, the composite broken specimen had an ARI value of 1, while the three ormocer broken specimens had an ARI value of 2. According to ARI scores, the layer of adhesive that was used, either composite or ormocer, mostly fractured cohesively.

Future research on this topic should include thermocycling of the samples and a higher number of samples. The settings for the mastication simulation, such as variable load and different lateral axes, should also be considered as additional variables.

### 4.1. Limitations and Strengths

One of our study’s limitations is the fact that it was an in vitro study, and another is that the mastication and bruxism conditions were simulated using a dual-axis chewing simulator. Also, we have used just one composite and one ormocer for every study group, and furthermore, each group included a limited number of teeth, using samples that were not thermocycled. We have used only one type of fixed retainer wire, namely 1.5-cm sections and pre-shaped multi-braided stainless steel wire, which was constructed into passive contact with the teeth’s lingual surface, because it is one of the most utilized fixed orthodontic retainers.

The strengths of this research come from creating a realistic model for simulating the natural mobility of teeth by using polyvinyl siloxane for periodontal ligament simulation. One of the main strengths is represented by the simulation of the periodontal tissues to faithfully reproduce intraoral conditions, assuring similar elasticity to natural teeth. Another strength is the use of a high magnification stereomicroscopy for sample micro-infiltration assertion at various magnifications, which ensured a three-dimensional rendering of the sample. Stereomicroscopy for evaluating the samples allowed us to measure and compare the values for the micro-infiltration with the total interface between the tested material and enamel. Not least, we have also tested an ormocer material, which has not been reported as an orthodontic fixed retainer bonding agent yet.

### 4.2. Future Directions

Future studies on a higher number of teeth and with increased material types in each category are encouraged. The comparison between bond strengths should be evaluated on round stainless steel wires, as well as on rectangular stainless steel wires, but also glass-fiber-reinforced composites. Also, along with one millimeter sections of the composite and ormocer samples, additional 0.5 mm samples could be investigated, for higher precision imaging. The authors agree that future research on this topic should include thermocycling of the samples and a higher number of samples. The settings for the mastication simulation, such as variable load and different lateral axes, should also be considered as additional variables.

## 5. Conclusions

Following careful consideration of the results and within the limitations of this study, we conclude that there is no significant difference between the mean value of micro-infiltration between the composite and ormocer specimens in the mastication group. For the bruxism group, the null hypothesis is rejected, revealing a statistical difference between the composite and ormocer specimens. The group statistics showed the mean value for the micro-infiltration in the composite (Gradia) specimens being more than double than the value of the ormocer (Admira) specimens. For the mastication group, one composite specimen suffered breakage during testing (ARI score 1), and for the bruxism groups three ormocer specimens (ARI score 2) and one composite specimen suffered breakage during testing (ARI score 1). For the purpose of strengthening clinical significance, more research on this topic is required.

## Figures and Tables

**Table 1:** Values of ratio between length of micro-infiltration and length of enamel-adhesive interface per 1 mm for each specimen, and material used.

Mastication Group and No. of Specimen	Ratio Value	Bruxism Group and No. of Specimen	Ratio Value
M_A1	0.0232	B_A1	0.1682
M_A2	0.1348	B_A2	0.2747
M_A3	0.0365	B_A3	0.0000
M_A4	0.0329	B_A4	0.0953
M_A5	0.0000	B_A5	Breakage after 7500 cycles
M_A6	0.1145	B_A6	0.0000
M_A7	0.0046	B_A7	Breakage after 10,000 cycles
M_A8	0.1765	B_A8	0.0709
M_A9	0.0000	B_A9	0.0563
M_A10	0.2490	B_A10	Breakage after 11,300 cycles
M_G1	0.3564	B_G1	0.2856
M_G2	0.0452	B_G2	0.2756
M_G3	0.3598	B_G3	0.1604
M_G4	0.7350	B_G4	Breakage after 8500 cycles
M_G5	0.2987	B_G5	0.4462
M_G6	0.0358	B_G6	0.0000
M_G7	Breakage after 45,000 cycles	B_G7	0.5520
M_G8	0.1973	B_G8	0.0932
M_G9	0.0239	B_G9	0.0000
M_G10	0.2270	B_G10	0.4002

**Table 2:** Group’s descriptive statistics.

	Ormocer Groups		Composite Groups	
	Mean	Std. * Deviation	Mean	Std. * Deviation
Mastication	0.096500	0.0869405	0.136313	0.1309995
Bruxism	0.253244	0.2245073	0.253244	0.2245073

*—Standard.

**Table 3:** Mann-Whitney U test for bruxism groups.

Test	Ratio Value
Mann–Whitney U	5.000
Wilcoxon W	20.000
Z-score	−2.030
Asymp. Sig. (2-tailed)	0.042
Exact Sig. [2 ∗ (1-tailed Sig.)]	0.048

Sig—significance; Z-score—standard score.

## Data Availability

Not applicable.
